# Amino­(5-{2-[amino­(iminio)meth­yl]hydrazin-1-yl}-3,5-dimethyl-4,5-dihydro-1*H*-pyrazol-1-yl)methaniminium dinitrate

**DOI:** 10.1107/S1600536810025006

**Published:** 2010-07-03

**Authors:** Sladjana B. Novaković, Mirjana Lalović, Vladimir Divjaković, Ljiljana S. Vojinović-Ješić, Valerija I. Češljević

**Affiliations:** a Vinča Institute of Nuclear Sciences, Laboratory of Theoretical Physics and Condensed Matter Physics, PO Box 522, 11001 Belgrade, Serbia; b Department of Chemistry, Faculty of Sciences, University of Novi Sad, Trg Dositeja Obradovića 3, 21000 Novi Sad, Serbia

## Abstract

The reaction of aqueous solutions of amino­guanidine hydrogennitrate and acetyl­acetone produces the title pyrazole salt, C_7_H_18_N_8_^2+^·2NO_3_^−^. The crystal structure is stabilized by a complex N—H⋯O hydrogen-bonding network. The difference in the engagement of the two nitrate anions in hydrogen bonding is reflected in the variation of the corresponding N—O bond lengths.

## Related literature

For the biological activity of pyrazole derivatives, see: Farag *et al.* (2008 ▶); Stauffer *et al.* (2000 ▶). For the coordination chemistry of pyrazole derivatives, see: Mukherjee (2000 ▶); Mani (1992 ▶). For related structures, see: Cousson *et al.* (1991*a* ▶,*b* ▶); Kettmann & Světlík (2002 ▶); Khudoyarov *et al.* (1995 ▶). For hydrogen-bonding motifs, see: Bernstein *et al.* (1995 ▶); Etter *et al.* (1990 ▶). Thiele & Dralle (1898 ▶) reported that the reaction of aqueous amino­guanidine hydrogennitrate and acetyl­acetone solutions led to the formation of acetyl­acetonebis(amino­guanidine) dihydrogendinitrate (C_7_H_16_N_8_·2HNO_3_). However, our investigations of the crystal and molecular structure of the obtained product have shown that this reaction did not form the cited Schiff base but a cyclic product of the same chemical composition.
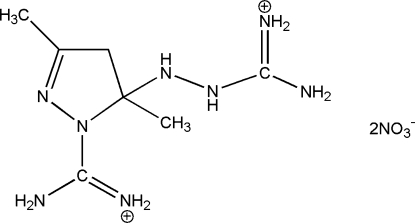


## Experimental

### 

#### Crystal data

C_7_H_18_N_8_^2+^·2NO_3_^−^
*M* *_r_* = 338.31
Orthorhombic, 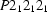

*a* = 7.5025 (2) Å
*b* = 13.8946 (4) Å
*c* = 14.2477 (3) Å
*V* = 1485.24 (7) Å^3^
*Z* = 4
Mo *K*α radiation
μ = 0.13 mm^−1^
*T* = 293 K
0.42 × 0.35 × 0.26 mm

#### Data collection

Oxford Diffraction Xcalibur Sapphire3 (Gemini Mo) diffractometer
4760 measured reflections
1997 independent reflections
1548 reflections with *I* > 2σ(*I*)
*R*_int_ = 0.017

#### Refinement

*R*[*F* ^2^ > 2σ(*F* ^2^)] = 0.043
*wR*(*F* ^2^) = 0.114
*S* = 1.03
1997 reflections
210 parameters
H-atom parameters constrained
Δρ_max_ = 0.42 e Å^−3^
Δρ_min_ = −0.33 e Å^−3^

### 

Data collection: *CrysAlis PRO* (Oxford Diffraction, 2008 ▶); cell refinement: *CrysAlis PRO*; data reduction: *CrysAlis PRO*; program(s) used to solve structure: *SHELXS97* (Sheldrick, 2008 ▶); program(s) used to refine structure: *SHELXL97* (Sheldrick, 2008 ▶); molecular graphics: *ORTEP-3* (Farrugia, 1997 ▶); software used to prepare material for publication: *WinGX* (Farrugia, 1999 ▶), *PLATON* (Spek, 2009 ▶) and *PARST* (Nardelli, 1983 ▶, 1995 ▶).

## Supplementary Material

Crystal structure: contains datablocks I, global. DOI: 10.1107/S1600536810025006/dn2584sup1.cif

Structure factors: contains datablocks I. DOI: 10.1107/S1600536810025006/dn2584Isup2.hkl

Additional supplementary materials:  crystallographic information; 3D view; checkCIF report

## Figures and Tables

**Table 1:** Hydrogen-bond geometry (Å, °)

*D*—H⋯*A*	*D*—H	H⋯*A*	*D*⋯*A*	*D*—H⋯*A*
N3—H3⋯O5^i^	0.86	2.52	3.048 (3)	120
N4—H4⋯O6^ii^	0.86	2.48	3.331 (5)	173
N5—H5*A*⋯O4	0.86	2.50	3.138 (4)	132
N5—H5*A*⋯O5	0.86	2.19	3.048 (4)	174
N5—H5*B*⋯O3^iii^	0.86	2.23	2.934 (3)	139
N6—H6*A*⋯O1^iv^	0.86	2.22	3.022 (3)	154
N6—H6*B*⋯O4^ii^	0.86	2.04	2.905 (4)	179
N7—H7*A*⋯O1	0.86	2.07	2.899 (3)	162
N8—H8*A*⋯O2	0.86	2.04	2.897 (3)	172
N8—H8*B*⋯O2^iii^	0.86	2.23	2.990 (3)	148

Symmetry codes: (i) 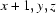; (ii) 
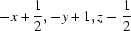
; (iii) 
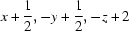
; (iv) 
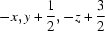
.

**Fig. 1.**
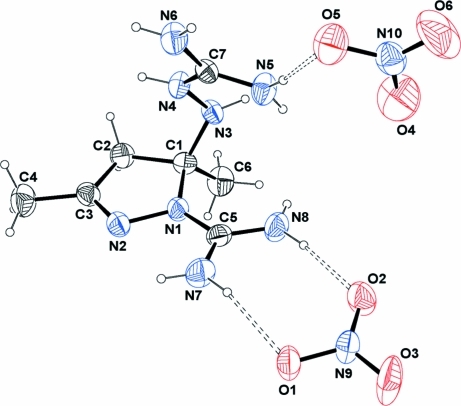
The molecular structure of (I), with atom labels and 50% probability displacement ellipsoids for non-H atoms. H atoms are represented as small spheres of arbitrary radii. Hydrogen bonds are shown as dashed lines.

**Fig. 2.**
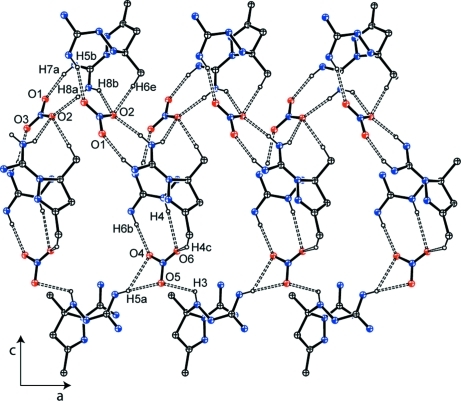
The packing diagram of (I), view approxymately normal to (010). H atoms not involved in hydrogen bonding have been omitted for clarity.

## supplementary crystallographic information

### Comment

In the paper (Thiele & Dralle, 1898) the reaction of aqueous aminoguanidine hydrogennitrate and acetylacetone solutions was described which, according to the authors, led to the formation of acetylacetonebis(aminoguanidine) dihydrogendinitrate (C_7_H_16_N_8_.2HNO_3_). However, our investigations of the crystal and molecular structure of the obtained product have shown that this reaction did not form the cited Schiff base but a cyclic product of the same chemical composition, *i.e.* amino(2-(1-(amino(iminio)methyl)-3,5-dimethyl-4,5-dihydro- 1*H*-pyrazol-5-yl)hydrazinyl)methaniminium-dinitrate (I).

Due to the presence of the nitrate anions next to the cation rich in N—H donor sites, the crystal structure of (I) (Figure 1) is stabilized by a very extensive hydrogen bonding network. The pair of the strongest hydrogen bonds (Table 1), N7—H7a···O1 and N8—H8a···O2, connects the protonated –C(NH_2_)_2_ substituent of the pyrazole ring to the single N9/O1/O2/O3 group generating an *R*^2^_2_(8) motif (Etter *et al.*, 1990; Bernstein *et al.*, 1995). The same nitrate group forms two additional hydrogen bonds (N5—H5b···O3 and N8—H8b···O2) that interlink the two –C(NH_2_)_2_ fragments of the pyrazolyl and hydrazinyl parts of the single molecule, producing the larger *R*^2^_2_(13) motif. These interactions, which are all shorter than 2.23 Å, generate a zigzag chain parallel to [100]. The hydrazinyl moiety of the cation also forms *R*^2^_2_(8) hydrogen bonding motif by engaging N4—H4 and N6—-H6b as donors to O6 and O4, respectively. In addition, the same nitrate anion (N10/O4/O5/O6) is involved in the bifurcated N5—H5a···O4 and N5–H5a···O5 hydrogen bond. The combination of these interactions extends the hydrogen bonding network toward [001] direction resulting in two-dimensional molecular arrays (Figure 2). This arrangement is also supported by two the strongest C—H···O interactions, while remaining N—-H6a···O1 and the weaker N—H···O and C—H···O interactions complete the three-dimensional structure. It is noteworthy that the nitrate group N9/O1/O2/O3 has the higher engagement in the strong hydrogen bonds (five hydrogen bonds < 2.23 Å) than N10/O4/O5/O6 (two hydrogen bonds < 2.23 Å). This is reflected in the corresponding N—O distances which in the first anion range from 1.212 (3)–1.269 (3) while in the second from 1.195 (4)–1.248 (3) Å. The oxygen atom of the shortest N—O6 bond engages only in weak N—H···O and C—H···O interactions.

### Experimental

To a solution of aminoguanidine hydrogennitrate (1.4 g, 10 mmol) in H_2_O (20 ml) acetylacetone (0.5 ml, 5 mmol) was added. The reaction mixture was homogenized by stirring on magnetic stirrer (20 min) at room temperature. After three days the resulting white crystals have been filtered and washed with water (35% yield).

### Refinement

The H atoms bonded to C and N atoms were placed at geometrically calculated positions and refined using a riding model. C—H distances were fixed to 0.96 and 0.97 Å from methyl and methylene C atoms respectively. Their *U*_iso_(H) values where equal to 1.5 times *U*_eq_ of the corresponding C (*sp*^3^) atom. N—H distances were fixed to 0.86 Å with *U*_iso_(H) values equal to 1.2 *U*_eq_ of the parent N.

In the absence of significant anomalous scattering, the absolute configuration could not be reliably determined and then the Friedel pairs were merged and any references to the Flack parameter were removed.

### Crystal data

**Table:**

C_7_H_18_N_8_^2+^·2NO_3_^−^	*F*(000) = 712
*M**_r_* = 338.31	*D*_x_ = 1.513 Mg m^−^^3^
Orthorhombic, *P*2_1_2_1_2_1_	Mo *K*α radiation, λ = 0.71073 Å
Hall symbol: P 2ac 2ab	Cell parameters from 2501 reflections
*a* = 7.5025 (2) Å	θ = 3.1–29.1°
*b* = 13.8946 (4) Å	µ = 0.13 mm^−^^1^
*c* = 14.2477 (3) Å	*T* = 293 K
*V* = 1485.24 (7) Å^3^	Prism, white
*Z* = 4	0.42 × 0.35 × 0.26 mm

### Data collection

**Table:**

Oxford Diffraction Xcalibur Sapphire3 (Gemini Mo) diffractometer	1548 reflections with *I* > 2σ(*I*)
Radiation source: fine-focus sealed tube	*R*_int_ = 0.017
graphite	θ_max_ = 29.2°, θ_min_ = 3.1°
Detector resolution: 16.3280 pixels mm^-1^	*h* = −10→7
ω scans	*k* = −16→17
4760 measured reflections	*l* = −19→19
1997 independent reflections	

### Refinement

**Table:**

Refinement on *F*^2^	Primary atom site location: structure-invariant direct methods
Least-squares matrix: full	Secondary atom site location: difference Fourier map
*R*[*F*^2^ > 2σ(*F*^2^)] = 0.043	Hydrogen site location: inferred from neighbouring sites
*wR*(*F*^2^) = 0.114	H-atom parameters constrained
*S* = 1.03	*w* = 1/[σ^2^(*F*_o_^2^) + (0.0719*P*)^2^] where *P* = (*F*_o_^2^ + 2*F*_c_^2^)/3
1997 reflections	(Δ/σ)_max_ < 0.001
210 parameters	Δρ_max_ = 0.42 e Å^−^^3^
0 restraints	Δρ_min_ = −0.33 e Å^−^^3^

### Special details

**Table:**

Geometry. All esds (except the esd in the dihedral angle between two l.s. planes) are estimated using the full covariance matrix. The cell esds are taken into account individually in the estimation of esds in distances, angles and torsion angles; correlations between esds in cell parameters are only used when they are defined by crystal symmetry. An approximate (isotropic) treatment of cell esds is used for estimating esds involving l.s. planes.
Refinement. Refinement of *F*^2^ against ALL reflections. The weighted *R*-factor *wR* and goodness of fit *S* are based on *F*^2^, conventional *R*-factors *R* are based on *F*, with *F* set to zero for negative *F*^2^. The threshold expression of *F*^2^ > σ(*F*^2^) is used only for calculating *R*-factors(gt) *etc*. and is not relevant to the choice of reflections for refinement. *R*-factors based on *F*^2^ are statistically about twice as large as those based on *F*, and *R*- factors based on ALL data will be even larger.

### Fractional atomic coordinates and isotropic or equivalent isotropic displacement parameters (Å^2^)

**Table:**

	*x*	*y*	*z*	*U*_iso_*/*U*_eq_	
N1	0.6023 (3)	0.23138 (16)	0.72769 (15)	0.0309 (5)	
N2	0.5967 (3)	0.19338 (16)	0.63655 (16)	0.0322 (5)	
N3	0.6670 (3)	0.39564 (15)	0.76413 (15)	0.0296 (5)	
H3	0.6855	0.4255	0.8161	0.036*	
N4	0.5571 (3)	0.43172 (16)	0.69281 (15)	0.0312 (5)	
H4	0.5944	0.4349	0.6359	0.037*	
N5	0.3293 (3)	0.44897 (18)	0.79956 (16)	0.0382 (6)	
H5A	0.2228	0.4676	0.8127	0.046*	
H5B	0.3938	0.4222	0.8421	0.046*	
N6	0.2960 (3)	0.50226 (18)	0.64889 (17)	0.0412 (6)	
H6A	0.1894	0.5212	0.6611	0.049*	
H6B	0.3394	0.5100	0.5935	0.049*	
N7	0.3393 (3)	0.15173 (18)	0.75060 (18)	0.0427 (6)	
H7A	0.2524	0.1339	0.7860	0.051*	
H7B	0.3423	0.1341	0.6928	0.051*	
N8	0.4640 (3)	0.23279 (18)	0.87330 (18)	0.0410 (6)	
H8A	0.3773	0.2151	0.9088	0.049*	
H8B	0.5481	0.2681	0.8956	0.049*	
C1	0.7481 (3)	0.30221 (18)	0.74174 (19)	0.0283 (5)	
C2	0.8406 (4)	0.2956 (2)	0.6454 (2)	0.0340 (6)	
H2A	0.8451	0.3581	0.6152	0.041*	
H2B	0.9610	0.2710	0.6517	0.041*	
C3	0.7278 (4)	0.22799 (19)	0.59131 (19)	0.0318 (6)	
C4	0.7588 (5)	0.2006 (2)	0.4917 (2)	0.0477 (8)	
H4A	0.6762	0.1511	0.4740	0.071*	
H4B	0.8784	0.1772	0.4846	0.071*	
H4C	0.7418	0.2559	0.4523	0.071*	
C5	0.4681 (3)	0.20601 (18)	0.78507 (19)	0.0306 (6)	
C6	0.8751 (4)	0.2760 (2)	0.8205 (2)	0.0426 (7)	
H6C	0.9766	0.3181	0.8188	0.064*	
H6D	0.9139	0.2106	0.8129	0.064*	
H6E	0.8153	0.2828	0.8797	0.064*	
C7	0.3929 (3)	0.46119 (18)	0.71523 (17)	0.0268 (5)	
N9	0.0699 (3)	0.09061 (19)	0.95468 (15)	0.0364 (6)	
O1	0.0933 (3)	0.05303 (16)	0.87444 (13)	0.0442 (5)	
O2	0.1651 (3)	0.15852 (17)	0.97976 (16)	0.0521 (6)	
N10	−0.0557 (4)	0.52379 (19)	0.92350 (18)	0.0418 (6)	
O3	−0.0469 (3)	0.0598 (3)	1.00527 (15)	0.0752 (9)	
O4	0.0522 (4)	0.4704 (3)	0.9632 (2)	0.0828 (9)	
O5	−0.0419 (3)	0.52961 (19)	0.83639 (16)	0.0562 (6)	
O6	−0.1669 (5)	0.5670 (2)	0.9663 (3)	0.1010 (12)	

### Atomic displacement parameters (Å^2^)

**Table:**

	*U*^11^	*U*^22^	*U*^33^	*U*^12^	*U*^13^	*U*^23^
N1	0.0339 (11)	0.0319 (11)	0.0268 (10)	−0.0079 (10)	0.0053 (10)	−0.0029 (10)
N2	0.0347 (11)	0.0323 (11)	0.0295 (10)	−0.0007 (10)	−0.0011 (11)	−0.0039 (10)
N3	0.0314 (11)	0.0302 (11)	0.0273 (10)	0.0030 (10)	−0.0032 (10)	−0.0059 (10)
N4	0.0311 (12)	0.0389 (12)	0.0238 (9)	0.0040 (11)	0.0021 (10)	0.0021 (10)
N5	0.0331 (11)	0.0514 (15)	0.0302 (10)	0.0120 (12)	0.0026 (11)	0.0029 (12)
N6	0.0418 (14)	0.0496 (14)	0.0323 (12)	0.0167 (13)	−0.0042 (10)	0.0003 (12)
N7	0.0363 (12)	0.0479 (13)	0.0440 (14)	−0.0148 (12)	0.0069 (12)	0.0011 (13)
N8	0.0413 (13)	0.0484 (14)	0.0334 (12)	−0.0106 (12)	0.0098 (12)	0.0002 (12)
C1	0.0249 (12)	0.0282 (11)	0.0318 (13)	−0.0012 (11)	−0.0016 (11)	−0.0002 (12)
C2	0.0293 (13)	0.0360 (14)	0.0365 (14)	0.0026 (12)	0.0068 (12)	−0.0006 (13)
C3	0.0342 (14)	0.0291 (12)	0.0320 (13)	0.0052 (12)	0.0005 (12)	−0.0022 (12)
C4	0.0491 (17)	0.0572 (19)	0.0367 (15)	0.0090 (17)	0.0088 (15)	−0.0050 (16)
C5	0.0320 (13)	0.0259 (12)	0.0338 (13)	0.0012 (11)	0.0035 (12)	0.0037 (11)
C6	0.0338 (15)	0.0535 (18)	0.0403 (16)	0.0079 (15)	−0.0081 (13)	0.0023 (15)
C7	0.0272 (12)	0.0267 (12)	0.0265 (11)	0.0001 (11)	−0.0026 (11)	−0.0047 (10)
N9	0.0317 (12)	0.0526 (15)	0.0249 (10)	−0.0043 (12)	0.0015 (11)	−0.0007 (11)
O1	0.0482 (11)	0.0567 (13)	0.0277 (9)	−0.0101 (11)	0.0064 (10)	−0.0083 (10)
O2	0.0543 (13)	0.0573 (13)	0.0448 (12)	−0.0216 (12)	0.0073 (11)	−0.0166 (12)
N10	0.0405 (14)	0.0442 (15)	0.0405 (13)	−0.0054 (13)	−0.0008 (12)	−0.0085 (12)
O3	0.0620 (15)	0.130 (3)	0.0335 (11)	−0.0500 (18)	0.0185 (12)	−0.0166 (15)
O4	0.094 (2)	0.097 (2)	0.0578 (15)	0.012 (2)	−0.0325 (17)	0.0030 (16)
O5	0.0557 (14)	0.0699 (15)	0.0430 (11)	−0.0017 (14)	−0.0097 (11)	0.0007 (12)
O6	0.092 (2)	0.0797 (19)	0.131 (3)	0.007 (2)	0.061 (2)	−0.035 (2)

### Geometric parameters (Å, °)

**Table:**

N1—C5	1.344 (3)	N8—H8B	0.8605
N1—N2	1.403 (3)	C1—C6	1.517 (4)
N1—C1	1.485 (3)	C1—C2	1.541 (4)
N2—C3	1.271 (4)	C2—C3	1.480 (4)
N3—N4	1.402 (3)	C2—H2A	0.9700
N3—C1	1.469 (3)	C2—H2B	0.9700
N3—H3	0.8601	C3—C4	1.488 (4)
N4—C7	1.336 (3)	C4—H4A	0.9600
N4—H4	0.8593	C4—H4B	0.9600
N5—C7	1.304 (3)	C4—H4C	0.9600
N5—H5A	0.8606	C6—H6C	0.9600
N5—H5B	0.8597	C6—H6D	0.9600
N6—C7	1.322 (3)	C6—H6E	0.9600
N6—H6A	0.8597	N9—O3	1.212 (3)
N6—H6B	0.8606	N9—O2	1.236 (3)
N7—C5	1.320 (4)	N9—O1	1.269 (3)
N7—H7A	0.8605	N10—O6	1.195 (4)
N7—H7B	0.8594	N10—O4	1.235 (4)
N8—C5	1.311 (4)	N10—O5	1.248 (3)
N8—H8A	0.8597		
			
C5—N1—N2	116.2 (2)	C3—C2—H2B	110.9
C5—N1—C1	130.1 (2)	C1—C2—H2B	110.9
N2—N1—C1	113.4 (2)	H2A—C2—H2B	109.0
C3—N2—N1	107.7 (2)	N2—C3—C2	114.8 (2)
N4—N3—C1	113.7 (2)	N2—C3—C4	120.5 (3)
N4—N3—H3	123.2	C2—C3—C4	124.7 (3)
C1—N3—H3	123.1	C3—C4—H4A	109.5
C7—N4—N3	118.6 (2)	C3—C4—H4B	109.5
C7—N4—H4	120.7	H4A—C4—H4B	109.5
N3—N4—H4	120.7	C3—C4—H4C	109.5
C7—N5—H5A	120.0	H4A—C4—H4C	109.5
C7—N5—H5B	120.0	H4B—C4—H4C	109.5
H5A—N5—H5B	120.0	N8—C5—N7	120.1 (3)
C7—N6—H6A	120.0	N8—C5—N1	121.7 (3)
C7—N6—H6B	120.1	N7—C5—N1	118.2 (2)
H6A—N6—H6B	120.0	C1—C6—H6C	109.5
C5—N7—H7A	120.0	C1—C6—H6D	109.5
C5—N7—H7B	120.0	H6C—C6—H6D	109.5
H7A—N7—H7B	120.0	C1—C6—H6E	109.5
C5—N8—H8A	120.0	H6C—C6—H6E	109.5
C5—N8—H8B	119.9	H6D—C6—H6E	109.5
H8A—N8—H8B	120.0	N5—C7—N6	120.9 (2)
N3—C1—N1	108.1 (2)	N5—C7—N4	121.2 (2)
N3—C1—C6	108.1 (2)	N6—C7—N4	117.9 (2)
N1—C1—C6	113.8 (2)	O3—N9—O2	121.0 (3)
N3—C1—C2	115.6 (2)	O3—N9—O1	119.3 (3)
N1—C1—C2	99.90 (19)	O2—N9—O1	119.6 (2)
C6—C1—C2	111.2 (2)	O6—N10—O4	121.7 (3)
C3—C2—C1	104.1 (2)	O6—N10—O5	122.2 (3)
C3—C2—H2A	110.9	O4—N10—O5	116.1 (3)
C1—C2—H2A	110.9		
			
C5—N1—N2—C3	177.1 (2)	N1—C1—C2—C3	3.4 (2)
C1—N1—N2—C3	3.2 (3)	C6—C1—C2—C3	123.9 (2)
C1—N3—N4—C7	129.5 (2)	N1—N2—C3—C2	−0.6 (3)
N4—N3—C1—N1	−61.2 (3)	N1—N2—C3—C4	179.7 (2)
N4—N3—C1—C6	175.2 (2)	C1—C2—C3—N2	−1.9 (3)
N4—N3—C1—C2	49.7 (3)	C1—C2—C3—C4	177.7 (3)
C5—N1—C1—N3	−55.7 (3)	N2—N1—C5—N8	176.2 (2)
N2—N1—C1—N3	117.2 (2)	C1—N1—C5—N8	−11.1 (4)
C5—N1—C1—C6	64.4 (4)	N2—N1—C5—N7	−2.9 (4)
N2—N1—C1—C6	−122.7 (3)	C1—N1—C5—N7	169.8 (2)
C5—N1—C1—C2	−176.9 (3)	N3—N4—C7—N5	−6.1 (4)
N2—N1—C1—C2	−4.1 (3)	N3—N4—C7—N6	174.6 (2)
N3—C1—C2—C3	−112.3 (2)		

### Hydrogen-bond geometry (Å, °)

**Table:**

*D*—H···*A*	*D*—H	H···*A*	*D*···*A*	*D*—H···*A*
N3—H3···O5^i^	0.86	2.52	3.048 (3)	120
N4—H4···O6^ii^	0.86	2.48	3.331 (5)	173
N5—H5A···O4	0.86	2.50	3.138 (4)	132
N5—H5A···O5	0.86	2.19	3.048 (4)	174
N5—H5B···O3^iii^	0.86	2.23	2.934 (3)	139
N6—H6A···O1^iv^	0.86	2.22	3.022 (3)	154
N6—H6B···O4^ii^	0.86	2.04	2.905 (4)	179
N7—H7A···O1	0.86	2.07	2.899 (3)	162
N8—H8A···O2	0.86	2.04	2.897 (3)	172
N8—H8B···O2^iii^	0.86	2.23	2.990 (3)	148

Symmetry codes: (i) *x*+1, *y*, *z*; (ii) −*x*+1/2, −*y*+1, *z*−1/2; (iii) *x*+1/2, −*y*+1/2, −*z*+2; (iv) −*x*, *y*+1/2, −*z*+3/2.

## References

[bb1] Bernstein, J., Davis, R. E., Shimoni, L. & Chang, N.-L. (1995). *Angew. Chem. Int. Ed. Engl.* **34**, 1555–1573.

[bb2] Cousson, A., Bachet, B., Kokel, B. & Hubert-Habart, M. (1991*a*). *Acta Cryst.* C**47**, 1885–1888.

[bb3] Cousson, A., Robert, F. & Hubert-Habart, M. (1991*b*). *Acta Cryst.* C**47**, 395–397.

[bb4] Etter, M. C., MacDonald, J. C. & Bernstein, J. (1990). *Acta Cryst.* B**46**, 256–262. 10.1107/s0108768189012929 2344397

[bb5] Farag, A. M., Mayhoub, A. S., Barakat, S. E. & Bayomi, A. H. (2008). *Bioorg. Med. Chem.* **16**, 881–889. 10.1016/j.bmc.2007.10.015 17962022

[bb6] Farrugia, L. J. (1997). *J. Appl. Cryst.* **30**, 565.

[bb7] Farrugia, L. J. (1999). *J. Appl. Cryst.* **32**, 837–838.

[bb8] Kettmann, V. & Světlík, J. (2002). *Acta Cryst.* C**58**, o423–o424. 10.1107/s010827010200937x 12094065

[bb9] Khudoyarov, A. B., Mirdzhalalov, F. F., Sharipov, Kh. T. & Khudaiberdyeva, S. P. (1995). *Uzb. Chem. J.* pp. 5–6.

[bb10] Mani, F. (1992). *Coord. Chem. Rev.* **120**, 325–359.

[bb11] Mukherjee, R. (2000). *Coord. Chem. Rev.* **203**, 151–218.

[bb12] Nardelli, M. (1983). *Comput. Chem.* **7**, 95–97.

[bb13] Nardelli, M. (1995). *J. Appl. Cryst.* **28**, 659.

[bb14] Oxford Diffraction (2008). *CrysAlis CCD* and *CrysAlis RED* Oxford Diffraction Ltd, Yarnton, England.

[bb15] Sheldrick, G. M. (2008). *Acta Cryst.* A**64**, 112–122. 10.1107/S0108767307043930 18156677

[bb16] Spek, A. L. (2009). *Acta Cryst.* D**65**, 148–155. 10.1107/S090744490804362X PMC2631630 19171970

[bb17] Stauffer, S. R., Coletta, C. J., Tedesco, R., Nishiguchi, G., Carlson, K., Sun, J., Katzenellenbogen, B. S. & Katzenellenbogen, J. A. (2000). *J. Med. Chem.* **43**, 4934–4947. 10.1021/jm000170m 11150164

[bb18] Thiele, J. & Dralle, E. (1898). *Annalen*, **302**, 275–334.

